# Posterior reversible encephalopathy syndrome in neuro-malaria

**DOI:** 10.4103/0971-3026.69357

**Published:** 2010-08

**Authors:** Alexis Lacout, Celine Guidoux, Robert Yves Carlier

**Affiliations:** 1 Department of Radiology, Centre d’imagerie Médicale, Centre Médico Chirurgical (CMC) – groupe VITALIA 83, Avenue Charles de, Gaulle 15000, Aurillac, France; 2 Service de réanimation médicale, hôpital Raymond Poincaré (assistance publique-hopitaux de Paris) 104 boulevard Raymond Poincaré, 92380 Garches, France; 3 Service de radiologie, hôpital Raymond Poincaré (assistance publique-hopitaux de Paris) 104 boulevard Raymond Poincaré, 92380 Garches, France

**Keywords:** Malaria, posterior reversible encephalopathy syndrome, Falciparum

## Abstract

We report a case of a 37-year-old patient with *Plasmodium falciparum* infestation who developed posterior reversible encephalopathy. In cerebral malaria, microscopic studies have shown endothelial dysfunction and disruption of the blood–brain barrier. Data from the literature show that one of the mechanisms of posterior reversible encephalopathy may be capillary leakage and acute disruption of the blood–brain barrier. Our case supports the theory of blood–brain barrier disruption being a key factor in the causation of cerebral malaria.

## Introduction

Our case is the first report of posterior reversible encephalopathy syndrome (PRES) in a patient with *Plasmodium falciparum* infestation, which could suggest a key role for blood–brain barrier disruption in the causation of cerebral malaria.

## Case History

A 37-year-old man was referred to our intensive care unit because of suspicion of malaria. He had returned to France after having stayed 7 weeks in Equatorial Guinea. Twelve days after his return he complained of fever, asthenia, headaches and myalgia. There was a decrease in think speed but no impairment of alertness and consciousness. There were no focal neurological signs or neck stiffness. Laboratory studies revealed a white cell count of 4600/mm ^3^(77% polynuclear neutrophils, 0.3% polynuclear eosinophils), hemoglobin level of 13 g/dl and a low platelet count of 15000/mm^3^. Liver enzyme levels were elevated, with an alanine aminotransferase level of 482 U/l, aspartate aminotransferase level of 572 U/l and alkaline phosphatase level of 105 U/l. Blood smears were positive for *P. falciparum*, with a high grade of parasitemia (40–50%)

Intravenous quinine dihydrochloride was administered but needed to be replaced 48 h later by artemisin because of hypoglycemia (1.9 mmol/l). At day 3 from admission the patient required mechanical ventilation and sedation because of acute respiratory distress syndrome. He was weaned off mechanical ventilation on day 17. The patient also developed transient acute renal failure and required hemodialysis. Neurological examination showed confusion, with disorientation and disturbances of memory, attention and concentration but without hallucination and agitation. There was no sensory deficit or pyramidal signs but there was bilateral severe weakness, which was ascribed to intensive therapy unit-acquired neuromyopathy. Electroencephalographic examination was normal. Blood pressure did not exceed 120/60 mm Hg during the period of hospitalization. One month later all neuropsychological tests were normal

**Figure 1 (A-C):**
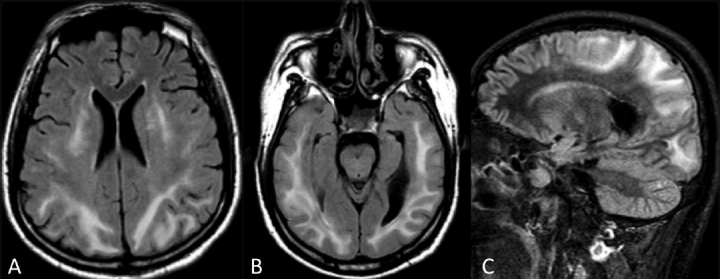
MRI of the brain in June, Axial FLAIR (A,B) and sagittal STIR-FLAIR (C) images show bilateral signal hyperintensity of the supratentorial white matter of the brain. The signal abnormalities have a predilection for the territories of the posterior circulation

Two MRI examinations of the brain were performed 2 months apart. On the first MRI examination, we observed diffuse symmetric signal-intensity abnormalities of the white matter of the posterior circulation territory, which consisted of subtle low-signal intensity on the T1W images and high intensity on the T2W, STIR-FLAIR and FLAIR images [Figure 1]. The gray matter of the cortex and basal ganglia were normal. Diffusion-weighted imaging (DWI) imaging showed subtle high-signal intensity and high apparent diffusion coefficient (ADC) values (1.000 10^-3^mm^2^/s in the posterior aspect of the temporal lobes, for example). We found subtle enhancement surrounding the lesions after injection of gadolinium. These imaging findings were consistent with predominantely posterior circulation white matter edema. We did not find any area of low or pseudonormalized ADC values consistent with cytotoxic edema.

**Figure 2 (A-C):**
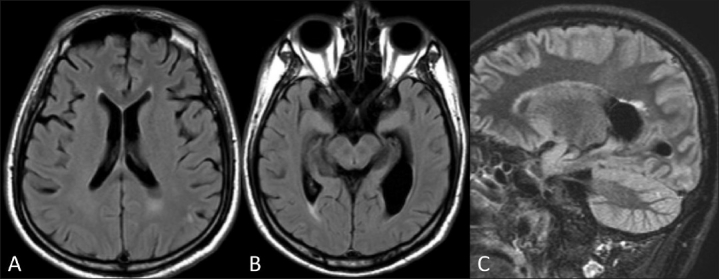
MRI of the brain in July. Axial FLAIR (A,B) and sagittal STIR-FLAIR (C) images show significant regression of the extent of the white matter involvement as well as the signal intensity

On the second MRI examination, we observed diminution of the extension of the white matter involvement as well as a diminution of the signal intensity abnormalities [Figure 2]. DWI imaging showed no regions of cytotoxic edema. These MRI findings were consistent with the diagnosis of PRES.

## Discussion

### Cerebral malaria

Cerebral malaria occurs in 2% of patients infected with *P. falciparum*.[1] In falciparum malaria disturbances of consciousness can be caused by systemic complications (fever or hypoglycemia, for example) and therefore, we required a strict definition of cerebral malaria. Thus, cerebral malaria was defined as a deep level of unconsciousness in the presence of *P. falciparum* asexual parasitemia and persisting after the correction of hypoglycemia and the exclusion of other encephalopathies. After a generalized convulsion, at least 6 h of coma in adults and 1 h in children are required to exclude a transient postictal state.[2] Our patient underwent an episode of hypoglycemia during the treatment. However, hypoglycemic encephalopathy involves the cortex and the basal ganglia, which were spared in our case.[3] Cerebral malaria must be distinguished from post-malaria neurologic syndrome (PMNS) where the acute onset of neurological or neuropsychiatric symptoms occurs after the parasitemia in patients recovering from *P. falciparum* infestation.[4] PMNS may be secondary to an immune process and may resemble acute disseminated encephalomyelitis (ADEM).[4]

The pathogenesis of cerebral malaria is not well known and several mechanisms have been suggested.[1,2] Firstly, the production of proinflammatory cytokines may cause cerebral toxicity. Secondly, capillaries are blocked by the adhesion of infested erythrocytes to the endothelium, the adherence of noninfested erythrocytes to infested erythrocytes and the adherence of infested erythrocytes to infested erythrocytes. The endothelium demonstrates disruption of the blood–brain barrier.[2] These factors could lead to vascular engorgement, reduction of the cerebral blood flow, edema and hypoxia.[1,2] Finally, Medana *et al*[5] suggested that cerebral malaria may be the consequence of disruption of axonal transport secondary to a broad range of cerebral insults induced by *P. falciparum* infestations. The histological findings in cerebral malaria include sequestration of infested erythrocytes mainly in cortical and perforating arteries, perivascular hemorrhages and white matter necrosis. Although edema is easy to identify in MRI studies, it is more difficult to document in postmortem histological studies[6] An MRI study of 24 patients revealed slightly increased cerebral volume but no sign of cerebral edema. The increased cerebral volume was attributed to increase in blood flow caused by vasodilatation and sequestration of infested erythrocytes[7] Another MRI study of cerebral malaria showed two types of brain involvement. In the first type, there were hyperintense cortical nodules on T2W images consistent with small cortical infarcts, presumably due to the blockage of capillaries by the infested erythrocytes.[6] In the second type there were white matter changes consistent with vasogenic edema[6] Sakai *et al*[1]. reported the presence of small white matter infarcts on MRI diffusion imaging in one patient with cerebral malaria.

### Posterior reversible encephalopathy syndrome

In our case the imaging findings were consistent with the diagnosis of PRES. PRES is a recently described syndrome where, classically, vasogenic edema involves primarily the white matter in the posterior circulation territories. Transformation to irreversible cytotoxic edema has been described[8] The cerebral edema may be either diffuse or relatively localized (e.g., isolated brain stem or basal ganglion involvement)[9] PRES may be observed in patients with hypertensive encephalopathy, eclampsia, renal failure, immunosuppressive treatments, thrombotic thrombocytopenia purpura and hemolytic uremic syndrome[10] PRES may also be a complication following infections[11–14]

### Cerebral malaria, PRES and the blood–brain barrier

The cause of PRES is not yet fully understood. Two main hypothesis are cited: 1) hypertension with hyperperfusion and 2) ischemia secondary to hypoperfusion[13,14] Immune activation, capillary leakage and acute disruption of the blood–brain barrier may also play a key role in the physiopathology of PRES[10]

PRES usually develops in patients with complex systemic conditions with multiple organ dysfunction syndrome (acute renal failure and acute respiratory distress syndrome in our case)[13,14] In this setting, hypoxemia (caused by hypoperfusion and toxic conditions) may result in strong vascular endothelial growth factor (VEGF) expression, which may favor endothelial activation and permeability and thus cause vasogenic edema[15]

In cerebral malaria, microscopic studies have shown endothelial dysfunction and disruption of the blood–brain barrier[2] *In vitro* studies showed that adhesion of infested erythrocytes to endothelial cells induces their apoptosis[16] Furthermore, *in vivo* studies in human and mice models of cerebral malaria shows that infiltrating cytotoxic CD8 T lymphocytes could lead to the death of the endothelial cells[16]

In our case the onset of PRES may have resulted from capillary leakage and disruption of the blood–brain barrier caused both by the complex, toxic systemic condition due to *P. falciparum* infestation and the blockage of the cerebral vessels by infested erythrocytes.

## Conclusion

Cerebral malaria is one of the most serious complications of *P. falciparum* infestation. Our patient developed PRES and this supports the hypothesis that disruption of the blood–brain barrier plays a key role in the causation of cerebral malaria in patients with *P. falciparum* infestation.
